# Short-Term Magnesium Deficiency Triggers Nutrient Retranslocation in *Arabidopsis thaliana*

**DOI:** 10.3389/fpls.2020.00563

**Published:** 2020-06-04

**Authors:** Takaaki Ogura, Natsuko I. Kobayashi, Christian Hermans, Yasunori Ichihashi, Arisa Shibata, Ken Shirasu, Naohiro Aoki, Ryohei Sugita, Takahiro Ogawa, Hisashi Suzuki, Ren Iwata, Tomoko M. Nakanishi, Keitaro Tanoi

**Affiliations:** ^1^Graduate School of Agricultural and Life Sciences, The University of Tokyo, Tokyo, Japan; ^2^Crop Production and Biostimulation Laboratory, Interfacultary School of Bioengineers, Université libre de Bruxelles, Brussels, Belgium; ^3^RIKEN BioResource Research Center, Tsukuba, Japan; ^4^RIKEN Center for Sustainable Resource Science, Yokohama, Japan; ^5^National Institute of Radiological Sciences, National Institutes for Quantum and Radiological Science and Technology, Chiba, Japan; ^6^Cyclotron and Radioisotope Center, Tohoku University, Sendai, Japan; ^7^Hoshi University, Tokyo, Japan; ^8^PRESTO, Japan Science and Technology Agency, Kawaguchi, Japan

**Keywords:** defense response, magnesium transporter, mineral profile, nutrient deficiency, photosynthesis, sucrose partitioning, translocation

## Abstract

Magnesium (Mg) is essential for many biological processes in plant cells, and its deficiency causes yield reduction in crop systems. Low Mg status reportedly affects photosynthesis, sucrose partitioning and biomass allocation. However, earlier physiological responses to Mg deficiency are scarcely described. Here, we report that Mg deficiency in *Arabidopsis thaliana* first modified the mineral profile in mature leaves within 1 or 2 days, then affected sucrose partitioning after 4 days, and net photosynthesis and biomass production after 6 days. The short-term Mg deficiency reduced the contents of phosphorus (P), potassium, manganese, zinc and molybdenum in mature but not in expanding (young) leaves. While P content decreased in mature leaves, P transport from roots to mature leaves was not affected, indicating that Mg deficiency triggered retranslocation of the mineral nutrients from mature leaves. A global transcriptome analysis revealed that Mg deficiency triggered the expression of genes involved in defence response in young leaves.

## Introduction

The magnesium ion (Mg^2+^) is the second most abundant cation in plant cells after potassium (Marschner, 1995) and is engaged in crucial biological functions for plants, such as stabilisation of chlorophyll (Strouse, 1974) and ribosome (Akanuma et al., 2014) structures and the regulation of enzymatic activities (Clarkson and Hanson, 1980; Cowan, 2002). Crop fertilisation most often focuses on nitrogen (N), phosphorus (P) and potassium (K), but ignores Mg (Fageria et al., 2008). Nonetheless, Mg deficiency affects the productivity of various crops (cereals, potatoes, sugar beets, etc.) and fruits (Gerendás and Führs, 2013).

The apparent symptom of Mg deficiency is interveinal chlorosis as a result of chlorophyll degradation (Marschner, 1995; Tanoi and Kobayashi, 2015). However, chlorophyll degradation is a late-stage symptom, because Mg bound to chlorophyll is not readily lost (Marschner, 1995). One of the earliest reported physiological symptoms is accumulation of sucrose and starch (Fischer and Bremer, 1993; Fischer et al., 1998; Hermans et al., 2004, 2005; Hermans and Verbruggen, 2005), due to impaired sucrose phloem loading from leaves (King and Zeevaart, 1974; Fischer and Bremer, 1993; Cakmak et al., 1994a, b; Hermans et al., 2005). Hermans and Verbruggen (2005) suggested that sucrose accumulation leads to the reduction in chlorophyll content through the repression of *CHLOROPHYLL A/B BINDING PROTEIN 2* (*CAB2*) gene. Cakmak and Kirkby (2008) proposed that sugar accumulation leads to chlorophyll degradation through the generation of reactive oxygen species (ROS); high sugar levels exert a negative feedback on photosynthesis (Sheen, 1994; Oswald et al., 2001), and the use of light energy for the photosynthetic electron transport chain decreases. This results in surplus light energy, which provides reducing equivalents with molecular oxygen to form ROS. Meanwhile, recent studies suggest that sugar accumulation is not the only cause of ROS generation, as the oxidative damage is detected in leaves of Mg-deficient rice before sugars accumulate (Kobayashi et al., 2013b, 2018) and roots of Mg-deficient sweet orange (Cai et al., 2019). Apart from that, a decrease in leaf transpiration is reported in Mg-deficient rice (Kobayashi et al., 2013b) and maize (Jezek et al., 2015), and this is suggested to be responsible for chlorosis development (Kobayashi et al., 2013b). To further clarify the mechanisms whereby Mg deficiency leads to chlorosis, it is important to identify early responses.

To cope with mineral deficiency, higher plants have strategies to optimise use and acquisition of deficient minerals. Retranslocation of minerals is the first strategy for plants to maintain growth during deficiency. This strategy requires mostly phloem transport of minerals from mature leaves. The mobility of Mg is highly variable among plant species, with retranslocation found in barley, bread wheat, rapeseed (Maillard et al., 2015), European ash (Hagen-Thorn et al., 2006) and Pedunculated oak (Hagen-Thorn et al., 2006; Maillard et al., 2015) but not in Arabidopsis (Himelblau and Amasino, 2001), maize, pea, black alder, black poplar (Maillard et al., 2015), silver birch or small-leaved lime (Hagen-Thorn et al., 2006). Mineral retranslocation is also induced during leaf senescence, and retranslocation of essential minerals during leaf senescence has been described in various plant species (Himelblau and Amasino, 2001; Waters and Grusak, 2008; Moreira and Fageria, 2009; Maillard et al., 2015). Meanwhile, studies on mineral deficiency mainly focus on retranslocation of the deficient mineral and rarely describe the profile of other minerals.

The second strategy is for roots to increase the uptake of deficient minerals. This strategy either induces the expression of root transporters (Gojon et al., 2009), stimulates the root growth (De Pessemier et al., 2013; Gruber et al., 2013) or exudes organic compounds that mobilise some minerals (Dakora and Phillips, 2002). The induction of root Mg^2+^ uptake is characterised in Arabidopsis (Ogura et al., 2018) and rice (Tanoi et al., 2014), and Mg^2+^ transporter family MITOCHONDRIA RNA SPLICING 2/MAGNESIUM TRANSPORTER (MRS2/MGT; Schock et al., 2000; Li et al., 2001) is indicated to be putatively responsible (Mao et al., 2014; Ogura et al., 2018). However, there is uncertainty regarding the root transcriptional regulation of *MRS2*/*MGT* genes with some reports suggesting that Mg deficiency induces root transcript levels of *MRS2*/*MGT* (Mao et al., 2014; Li et al., 2017; Liu et al., 2019) and others suggesting no transcriptional induction of *MRS2/MGT* (Gebert et al., 2009; Hermans et al., 2010a, b; Ogura et al., 2018).

The aims of the current study were firstly to identify the physiological and transcriptional responses to Mg deficiency prior to the reported physiological symptoms such as sugar accumulation. Since the symptoms of mineral deficiency usually appear on leaves at a specific age, leaf-specific responses were investigated in Arabidopsis. Secondly, the mineral profile of Mg-deficient plants was investigated for possible retranslocation of Mg and other minerals. Finally, the effects of Mg deficiency on root Mg^2+^ uptake and the expression of putative Mg^2+^ transporters in roots were re-examined.

## Materials and Methods

### Hydroponic Culture System

**FIGURE 1:**
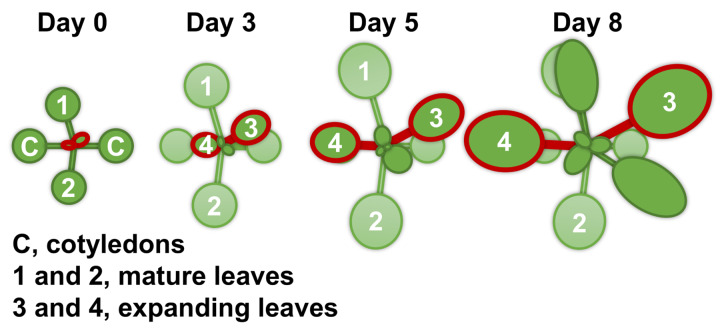
A schematic diagram of the shoot growth of *Arabidopsis thaliana* under the control condition (magnesium 1.5 mM). The Columbia-0 accession was grown hydroponically at 22°C and under a photoperiod of 16 h light (100 μmol m^–2^ s^–1^)/8 h darkness. Ten days after germination (day 0 of treatment), half of the plants were subjected to Mg deficiency (no Mg added). Mature leaves are identified as the first two leaves 1 and 2, and expanding leaves as 3 and 4.

For all measurements, with the exception of root morphology analysis, *Arabidopsis thaliana* ecotype Columbia-0 (Inplanta Innovations Inc., Yokohama, Japan) was grown hydroponically. Three to four seeds were placed on a piece of soaked polyurethane sponge (KP-1300, Hikari, Osaka, Japan) 5 mm in thickness. Up to 32 sponges were set into a float and placed in a container with 2 L of a hydroponic solution. The containers were covered with plastic wrap to make a high humidity environment for germination and placed in a growth room at 22°C and under a photoperiod of 16 h light (100 μmol m^–2^ s^–1^)/8 h darkness. After 1 week, the plastic wrap was removed, and the seedlings were thinned to leave one seedling per sponge. Ten days after germination, half of the plants were subjected to Mg deficiency treatment. At this point (day 0), the size of the first two rosette leaves was similar to that of the cotyledons, and the third and fourth rosette leaves had just appeared. We refer to the first two rosette leaves and the next two rosette leaves as mature leaves and expanding leaves, respectively (Figure 1). After the onset of Mg deficiency treatment, the fresh shoots and roots were separately weighed for four plants every 2 days, and the shoot-to-root fresh weight ratio was calculated. The two expanding leaves of each plant were weighed for eight plants every day from day 2. For the hydroponic solution, we used the MGRL solution (Fujiwara et al., 1992), which contained 1.5 mM MgSO_4_, 3.0 mM KNO_3_, 2.0 mM Ca(NO_3_)_2_, 1.5 mM NaH_2_PO_4_, 0.25 mM Na_2_HPO_4_, 30 μM H_3_BO_3_, 10 μM MnSO_4_, 8.6 μM FeSO_4_, 0.13 μM CoCl_2_, 1.0 μM CuSO_4_, 1.0 μM ZnSO_4_, 0.024 μM (NH_4_)_6_Mo_7_O_24_ and 67 μM EDTA-2Na (pH 5.8). For the Mg deficiency treatment, 1.5 mM MgSO_4_ in the original MGRL solution was substituted with 1.5 mM Na_2_SO_4_ to compensate for the lack of sulphate. All the hydroponic solutions were renewed every 2 days.

### Chlorophyll Content Analysis

Chlorophyll content in expanding leaves was determined for eight plants. Leaves were flash-frozen with liquid nitrogen, and chlorophyll was extracted with 250 μL of 80% (v/v) acetone using a bead homogeniser (BMS-A20TP, Bio Medical Science, Tokyo, Japan). The extract was left overnight at 4°C and centrifuged at 14,000 *g* for 10 min. The supernatant was analysed for its optical densities by a UV-Vis spectrophotometer (NanoDrop, Thermo Fisher Scientific, Waltham, MA, United States) at 647 and 664 nm. The total content of chlorophylls *a* and *b* in the extract was determined by including two optical density values in the equations described by Porra et al. (1989).

### Determination of Carbon Assimilation and Photosynthate Partitioning

The carbon dioxide (CO_2_) assimilation in leaves and subsequent photosynthate partitioning were evaluated radiometrically using radiolabelled CO_2_ (^14^CO_2_), as previously described by Sugita et al. (2018). The ^14^CO_2_ molecules (0.2 MBq) were generated in a vial by the chemical reaction between ^14^C-sodium bicarbonate (200 kBq, PerkinElmer, Waltham, MA, United States) and lactic acid. At 12 h into the light period, four plants from each treatment were supplied with ^14^CO_2_ for 15 min at 22°C by sending air from the vial to an airtight polypropylene bag containing all plants. Immediately after the ^14^CO_2_ introduction, all leaves and roots were separated and contacted to an Imaging Plate (BAS-IP MS, GE Healthcare, Buckinghamshire, United Kingdom) at 4°C. The Imaging Plate was scanned by a laser scanner (FLA-5000, GE Healthcare, Buckinghamshire, United Kingdom), and ^14^C in each part of the plant was quantified with an image analysis software (Image Gauge, Fujifilm, Tokyo, Japan). The CO_2_ assimilation rate was determined after dividing the amount of ^14^C assimilated in leaves for 15 min by the leaf area.

### Sugar Quantification

The sucrose and glucose contents in expanding leaves were determined enzymatically according to Okamura et al. (2016). Expanding leaves were harvested and composited from two to five plants (ca. 10 mg fresh weight) at 3 h into the light period. The composited sample was flash-frozen with liquid nitrogen and powdered with a bead homogeniser (BMS-A20TP, Bio Medical Science, Tokyo, Japan). Soluble sugars were successively extracted by double extraction using 500 and 200 μL of 80% (v/v) ethanol at 80°C for 10 min each time. After evaporation, samples were resuspended in 250 μL of distilled water using an ultrasonic bath (Branson 1200, Yamato Scientific, Tokyo, Japan). The contents of sucrose and glucose in solution were quantified with the F-kit #716260 (J. K. International, Tokyo, Japan).

### Elemental Analysis

The contents of mineral elements were determined in mature leaves, expanding leaves and roots. The fresh tissue samples were harvested from four plants, weighed separately and dried overnight at 60°C. The dried sample was digested with 2 mL of 30% (v/v) HNO_3_ (Kanto Chemical, Tokyo, Japan) at 90°C for 1 h. The digested solution was diluted 30× with Milli-Q water (Merck Millipore, Burlington, MA, United States) and filtered before the elemental analysis. The inductively coupled plasma-mass spectrometer NexION 350S (PerkinElmer, Waltham, MA, United States) was used to determine the contents of Mg, P,K,calcium (Ca), boron (B), manganese (Mn), copper (Cu), zinc (Zn) and molybdenum (Mo) in the digested solution.

### Determination of Phosphorus Transport

The process of P transport from roots to leaves was evaluated radiometrically with radiolabelled phosphate (^32^P-phosphate), as previously described by Sugita et al. (2017). The ^32^P-phosphate (PerkinElmer, Waltham, MA, United States) was added to each treatment solution (6 MBq L^–1^; phosphate, 1.75 mM) and introduced into four plants by soaking their roots for 30 min at 22°C under light conditions (100 μmol m^–2^ s^–1^). Roots were subsequently rinsed for 10 min with an ice-cold MGRL solution to wash out ^32^P from the apoplast. Phosphorus transported into mature and expanding leaves was separately quantified using an Imaging Plate (BAS-IP MS, GE Healthcare, Buckinghamshire, United Kingdom), as previously described by Kobayashi et al. (2013a).

### Global Transcriptome Analysis

The transcriptome was analysed with RNA sequencing (RNA-seq, Ozsolak and Milos, 2011). Mature leaves, expanding leaves and roots were separated, and tissues from two or three plants (ca. 5–30 mg fresh weight) were composited. A protocol of Breath Adapter Directional sequencing (BrAD-seq; Townsley et al., 2015; Ichihashi et al., 2018) was used to construct a strand-specific RNA-seq library, with which the entire transcriptome can be analysed including vital non-processed RNAs (Mills et al., 2013). Briefly, six tissue samples were lysed with a lysis/binding buffer and mRNA was extracted from the lysate. The quality of RNA was assessed with an Agilent 2100 Bioanalyzer and at least three biological replicates were selected for further analysis. After mRNA was fragmented and primed with 3′ adapter, cDNA was synthesised and strand-specific 5′ adapter sequence was added and incorporated. The average fragment length of the constructed library was approximately 400 bp. All the libraries were pooled and subjected to 50 bp single-end sequencing with Illumina HiSeq 2500 by Macrogen Japan (Kyoto, Japan).

The obtained reads were analysed according to Ichihashi et al. (2018). Briefly, the reads were mapped to the TAIR10 Arabidopsis reference genome sequence after removing low-quality reads. The mapped reads were counted, and the count data were normalised. The raw reads and normalised count data have been deposited in NCBI’s Gene Expression Omnibus (Edgar et al., 2002) and are accessible through GEO Series accession number GSE140070. Changes in gene expression by Mg deficiency were determined as the logarithm of A/B to the base two, with A and B a normalised count of Mg-deficient plants and that of control plants, respectively. Thereafter, the statistical analysis identified the differentially expressed genes (DEGs) during deficiency by using the Exact Test (*p* < 0.01, Robinson and Smyth, 2008) incorporated in edgeR Bioconductor package (Robinson et al., 2010). The up- and down-regulated genes were subjected to gene ontology (GO) enrichment analysis. The GO enrichment was determined by PANTHER Overrepresentation Test, using GO Ontology database released on the 6th of September 2018 for the annotation version, GO biological process complete for the annotation dataset, and the Fisher’s exact test with a false discovery rate (FDR) correction (*p* < 0.05) for the test type.

### Determination of Root Magnesium Uptake

The root Mg^2+^ uptake rate was determined radiometrically, as previously described by Tanoi et al. (2013). The radioisotope ^28^Mg was produced with ^27^Al(α, 3p)^28^Mg reaction in a cyclotron and was purified following the procedure of Iwata et al. (1992). Four plants from each treatment were fed with 1/30-strength MGRL solution containing ^28^Mg (6 MBq L^–1^; Mg^2 +^, 50 μM) for 1 h at 22°C under light conditions (100 μmol m^–2^ s^–1^). Roots were subsequently rinsed for 10 min with an ice-cold MGRL solution to wash out ^28^Mg from the apoplast. After harvest, the whole plant tissue was solubilised with 0.5 mL of Soluene^®^ -350 (PerkinElmer, Waltham, MA, United States) and mixed with 3 mL of liquid scintillation cocktail (Hionic-Fluor, PerkinElmer, Waltham, MA, United States). Magnesium taken up by each plant was quantified using a liquid scintillation counter (LSC-6100, Aloka, Tokyo, Japan), as previously described by Sugita et al. (2013). Since the ^28^Mg half-life is ca. 21 h and its radioactivity decreased while all the samples were analysed, the activity at the same time point was calculated for all the samples.

### Root Morphology Analysis

The morphology of the root system was examined for the wild type Columbia-0 and the following transfer DNA (T-DNA) insertion lines of *MRS2*/*MGT* genes: single mutant lines of *MRS2-4*/*MGT6*, *MRS2-1*/*MGT2* and *MRS2-10*/*MGT1*, and the three double and one triple mutant lines of *MRS2* genes that belong to the MRS2 subclade B (Gebert et al., 2009), namely *MRS2-1*/*MGT2*, *MRS2-5*/*MGT3* and *MRS2-10*/*MGT1*. Seeds of *mrs2-4* (SALK_145997) were provided by Dr. T. Kamiya (Oda et al., 2016), and seeds of *mrs2-1* (SALK_006797C), *mrs2-10* (SALK_100361C), *mrs2-1* × *mrs2-5*, *mrs2-1* × *mrs2-10*, *mrs2-5* × *mrs2-10* and *mrs2-1* × *mrs2-5* × *mrs2-10* lines were provided by Dr. V. Knoop (Gebert et al., 2009; Lenz et al., 2013). Seeds were surface sterilised with 70% (v/v) ethanol for 10 min and 20% (v/v) HClO solution for 5 min, and rinsed twice with sterile water. Plant growth conditions were as described in Xiao et al. (2015). Briefly, seeds were plated on 1× strength Murashige and Skoog medium (Murashige and Skoog, 1962) modified to contain a single source and lower concentration of nitrogen (10 mM KNO_3_), 1% (w/v) sucrose, 0.8% (w/v) agar (high gel strength agar, A9799, Sigma-Aldrich, St. Louis, MO, United States) and a final pH of 5.7. Control or low Mg plates contained 1,500 μM MgSO_4_ or 5 μM MgSO_4_ + 1,495 μM Na_2_SO_4_, respectively. Seeds were stratified at 4°C for 2 days and incubated in a growth chamber at 22°C and under a photoperiod of 16 h light (100 μmol m^–2^ s^–1^)/8 h darkness. Ten days after germination, root morphology parameters were measured for 13–38 seedlings, as described in Xiao et al. (2015), and fresh root and shoot organs were separately weighed.

### Statistical Analysis

The Student’s *t*-test was carried out using Microsoft Excel version 1909 to compare differences in the measured variables between control and Mg-deficient plants. A factorial analysis of variance (ANOVA) was used to test differences of the measured variables in the root morphology analysis. When a difference was found, the Tukey-Kramer test was carried out using R software version 3.6.0 to compare the values of different lines and to test in which comparison the difference was significant. Significance was set at the 5% level.

## Results

### Growth and Leaf Chlorophyll Content Were Affected After Six Days of Magnesium Deficiency

**FIGURE 2:**
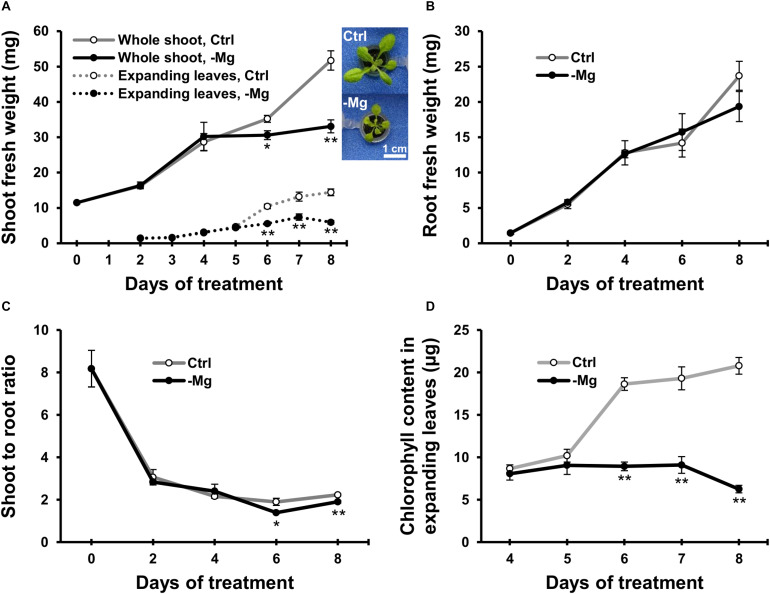
Effect of magnesium deficiency on biomass production and chlorophyll content. **(A)** Evolution of the fresh weight of the whole shoot (solid lines, *n* = 4) and expanding leaves (dotted lines, *n* = 8). The inserted images are the representative plant phenotypes on day 8 of control and Mg deficiency treatment. Scale bar: 1 cm. **(B)** Evolution of the fresh weight of root organs (*n* = 4). **(C)** The shoot-to-root ratio on the basis of fresh weight (*n* = 4). **(D)** The chlorophyll content in expanding leaves (*n* = 8). Data represent means with standard error. Ctrl, control; -Mg, magnesium deficiency. Asterisks indicate significant differences in measured variables between control and Mg-deficient plants (Student’s *t*-test, *p < 0.05, **p < 0.01).

To characterise early responses to Mg deficiency, we cultivated wild-type Arabidopsis plants hydroponically for 10 days with a Mg-replete solution (1.5 mM) and then fed half of the plants with a Mg-deplete (0 mM) solution. Fresh biomass production of the whole shoot, expanding leaves and root was measured during 8 days of treatment. In control plants, the fresh weight of the whole shoot and expanding leaves gradually increased (Figure 2A). In Mg-deficient plants, the biomass production was affected on day 6, when the fresh weight of the whole shoot and expanding leaves were 13% (*p* < 0.05) and 44% (*p* < 0.01) lower, respectively, compared to the control plants (Figure 2A). However, there was no difference in the root fresh weight between Mg conditions until day 8 (Figure 2B). Therefore, the shoot-to-root fresh weight ratio was lower (*p* < 0.05) in Mg-deficient plants from day 6 (Figure 2C).

Chlorophyll content was measured in expanding leaves between days 4 and 8, when the plants exhibited strong growth inhibition due to Mg deficiency. The chlorophyll content was unaffected until day 5 and decreased thereafter during Mg deficiency compared to the control plants (Figure 2D). On day 6, the chlorophyll content in Mg-deficient leaves was 52% lower (*p* < 0.01) compared to the control leaves (Figure 2D). Therefore, the time window of chlorophyll reduction overlapped with the reduction in shoot fresh weight.

### Carbon Assimilation and Photosynthate Partitioning Were Affected After Four to Five Days of Magnesium Deficiency

**FIGURE 3:**
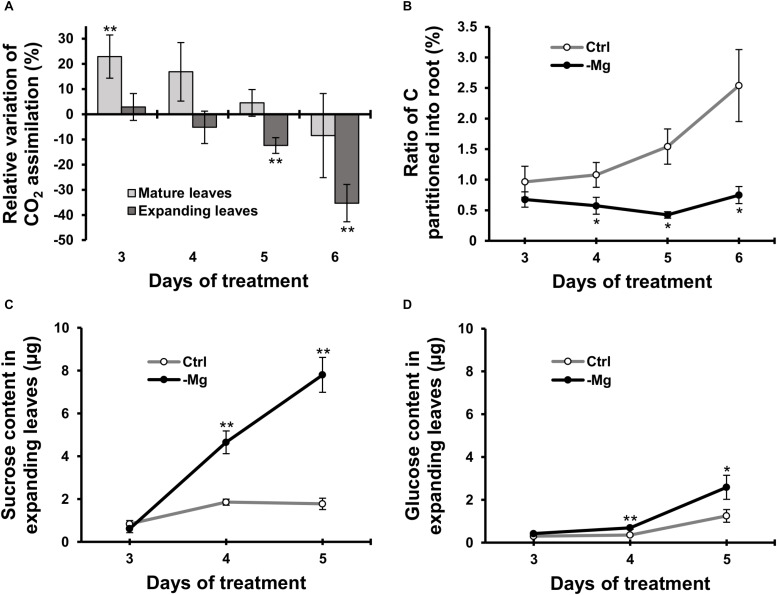
Effect of magnesium deficiency on carbon assimilation and photosynthate partitioning (^14^CO_2_ tracer study). **(A)** Relative variation of carbon assimilation rate by magnesium deficiency (^14^C tracer study, *n* = 4). The relative variation was determined after dividing the difference in the amount of ^14^C assimilated in mature leaves (pale grey) or expanding leaves (dark grey) between control and Mg-deficient plants by the amount of assimilated ^14^C in control plants. **(B)** The ratio of photosynthate partitioned into roots (^14^C tracer study, *n* = 4). The ratio was determined after dividing the amount of ^14^C in roots by the amount of ^14^C in the whole plant. **(C,D)** The contents of sucrose **(C)** and glucose **(D)** in expanding leaves determined enzymatically (*n* = 6). Data represent means with standard error. Ctrl: control, -Mg: magnesium deficiency. Asterisks indicate significant differences in measured variables between control and Mg-deficient plants (Student’s *t*-test, *p < 0.05, **p < 0.01).

We used ^14^C-labelled CO_2_ as a tracer to determine the CO_2_ assimilation rate in leaves between days 3 and 6, when the chlorophyll content started to decrease due to Mg deficiency. In mature leaves, the CO_2_ assimilation rate was greater (*p* < 0.01) in Mg-deficient than in control plants on days 3, and then it was similar between control and Mg-deficient plants until day 6 (Figure 3A). In expanding leaves, the CO_2_ assimilation rate was lower in Mg-deficient plants from day 5 (Figure 3A), which is before the reduction in fresh weight and chlorophyll content. On day 5, the CO_2_ assimilation rate in expanding leaves was 12% lower (*p* < 0.05) compared to the control plants (Figure 3A).

To evaluate photosynthate partitioning from source leaves to roots, we determined the ratio of ^14^C partitioned into the root to the total ^14^C in the plant. The ratio was similar between control and Mg-deficient plants on day 3 (Figure 3B). After day 3, the ratio gradually increased in control plants, whereas it was constant in Mg-deficient plants (Figure 3B). On day 4, the ratio was 47% lower (*p* < 0.05) in Mg-deficient than in control plants (Figure 3B). The disruption in photosynthate partitioning was detected at an early stage of Mg deficiency (day 4) when the CO_2_ assimilation was not inhibited yet. Consistently, the contents of sucrose and glucose in expanding leaves were higher in Mg-deficient than in control plants from day 4 (Figures 3C,D). On day 4, the sucrose and glucose contents were 3.0 (*p* < 0.01) and 2.3 (*p* < 0.01) times greater, respectively, in Mg-deficient plants (Figures 3C,D).

### Mineral Contents Were Reduced in Mature Leaves Within Two Days of Magnesium Deficiency

**FIGURE 4:**
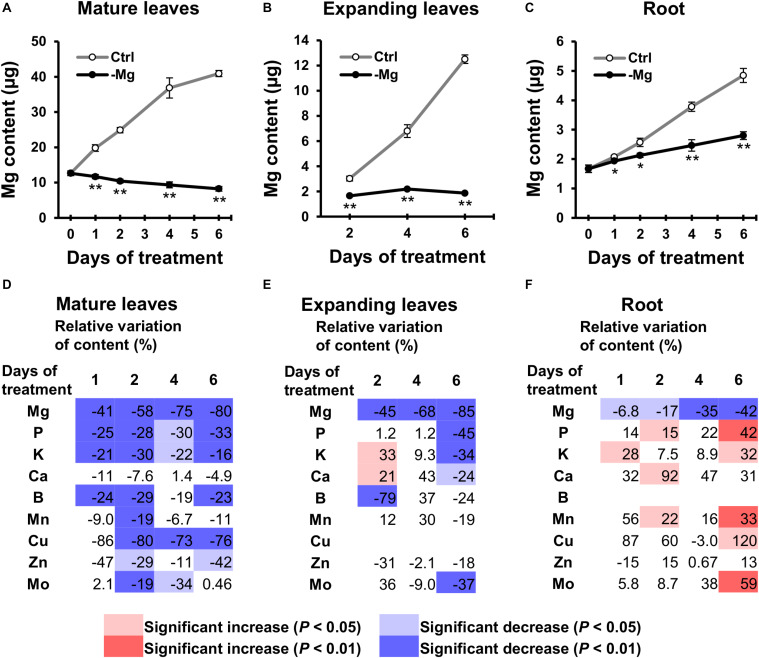
Effect of magnesium deficiency on the plant mineral profile. **(A–C)** The magnesium content in mature leaves **(A)**, expanding leaves **(B)** and roots **(C)** during magnesium deficiency. Data represent means (*n* = 4) with standard error. Ctrl, control; -Mg, magnesium deficiency. Asterisks indicate significant differences in the Mg content between control and Mg-deficient plants (Student’s *t*-test, *p < 0.05, **p < 0.01). **(D–F)** Relative variation of mineral content in mature leaves **(D)**, expanding leaves **(E)** and roots **(F)** during magnesium deficiency. The relative variation was determined after dividing the difference in mineral contents between control and Mg-deficient plants by the mineral content in control plants. The contents of copper (Cu) in expanding leaves and boron (B) in roots are not represented, because they were below the detection limit of ICP-MS measurement. Data represent means (*n* = 4). The cell colours indicate significant differences in the mineral contents between control and Mg-deficient plants (Student’s *t*-test, pale colours, *p* < 0.05; dark colours, *p* < 0.01).

To examine changes in the mineral profile, mineral contents in mature leaves and root were determined on days 0, 1, 2, 4 and 6, and contents in expanding leaves were determined on days 2, 4 and 6. The Mg content increased in control plants along with the growth in all organs analysed (Figures 4A–C). By contrast in Mg-deficient plants, the Mg content in mature leaves gradually decreased, the content in expanding leaves remained constant, and the content in root slowly increased (Figures 4A–C). Compared to the control plants, the Mg content was 41% (*p* < 0.01) and 6.8% (*p* < 0.05) lower in mature leaves and root on day 1, respectively, and 58% (*p* < 0.01), 45% (*p* < 0.01) and 17% (*p* < 0.05) lower in mature leaves, expanding leaves and root on day 2, respectively (Figures 4A–F), which indicates that the loss of Mg was more severe in the shoot than in the root.

For the other minerals, with the exception of Ca, the general tendency showed the relative decrease in mineral contents in mature leaves and no change in contents in expanding leaves and root, compared to the control plants. For instance, the P content in mature leaves was lower (*p* < 0.05) in Mg-deficient than in control plants between days 1 and 6 (Figure 4D), while the content in expanding leaves was maintained until day 4 (Figure 4E), and the content in root was sometimes even higher (*p* < 0.05) in Mg-deficient plants (Figure 4F). The relative decrease in mineral contents in mature leaves within 2 days of Mg deficiency was observed for P,K,B,Mn,Cu,Zn and Mo (Figure 4D). Some of these minerals (P,K,Mn,Zn and Mo) showed no change or an increase in contents in expanding leaves and root before day 6 (Figures 4E,F). On day 6, contents of P,K,Ca and Mo in expanding leaves were lower (*p* < 0.05) in Mg-deficient than in control plants (Figure 4E), which corresponds to the relative decrease in biomass (Figure 2A).

### Phosphorus Transport From Roots to Mature Leaves Was Not Affected During Six Days of Magnesium Deficiency

**FIGURE 5:**
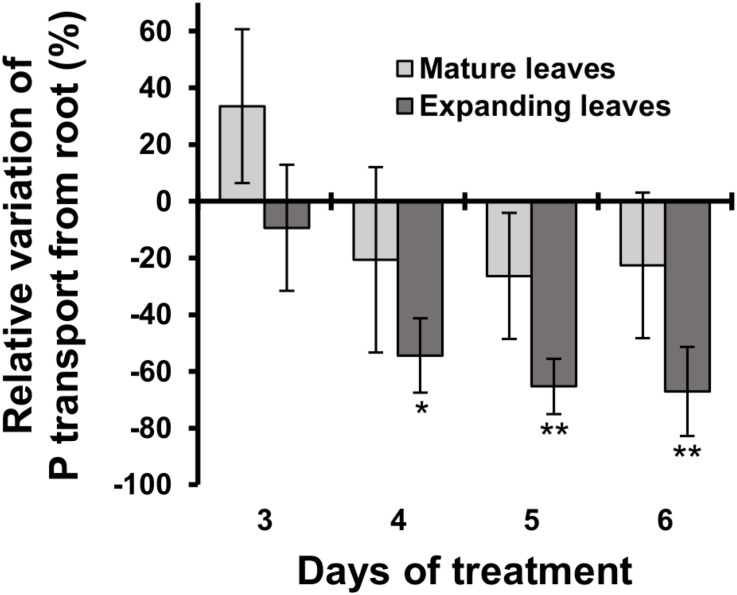
Effect of magnesium deficiency on short-term phosphorus transport (^32^P tracer study). Relative variation of P transport from roots by Mg deficiency was determined after dividing the difference in the amount of P transported into mature leaves (pale grey) or expanding leaves (dark grey) between control and Mg-deficient plants by the amount of transported P in control plants. Data represent means (*n* = 4) with standard error. Asterisks indicate significant differences in the amount of transported P between control and Mg-deficient plants (Student’s *t*-test, *p < 0.05, **p < 0.01).

The relative decrease in mineral contents in mature leaves could be attributed to an inhibition of root uptake and xylem transport from roots to mature leaves during Mg deficiency. To test that possibility, we determined the short-term transport of P from roots to leaves between days 3 and 6 using ^32^P-labelled phosphate as a radiotracer. Between days 3 and 6, the short-term P transport into mature leaves was not affected significantly (*p* ≥ 0.05) by Mg deficiency, but there was a numerical decrease (*p* < 0.05) in P transport into mature leaves between days 4 and 6 (Figure 5), which can be partly responsible for the reduced P content in mature leaves. On day 3, however, the P transport into mature leaves was slightly (but not significantly) more active in Mg-deficient plants (Figure 5). This excludes the possibility that the inhibition of root uptake and xylem transport is the cause of reduced P content in mature leaves within 2 days of Mg deficiency.

The P transport into expanding leaves was unaffected on day 3 and decreased thereafter during Mg deficiency compared to the control plants (Figure 5). The P transport into expanding leaves of Mg-deficient plants was 54% lower (*p* < 0.05) compared to that of control plants on day 4 (Figure 5), when the P content in expanding leaves was similar between control and Mg-deficient plants (Figure 4E). This suggests that the reduced P transport from roots to expanding leaves was balanced by retranslocation from other plant parts.

### Transcriptome Analysis Revealed Leaf Position-Specific and Time-Dependent Gene Expression Profile During Magnesium Deficiency

**FIGURE 6:**
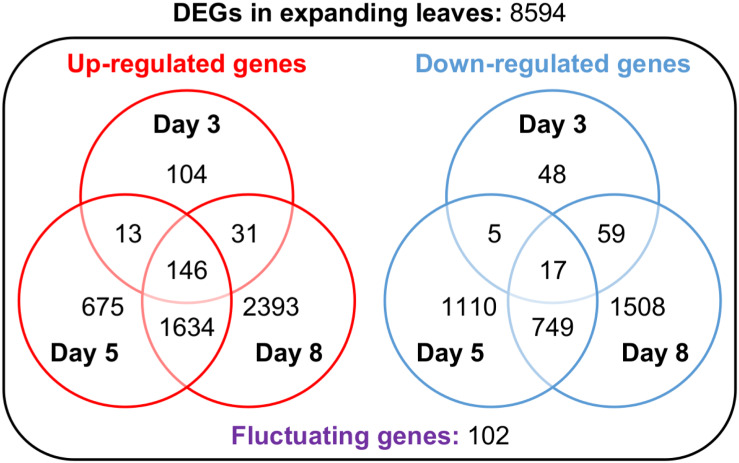
Venn diagrams representing the number of genes up- or down-regulated in expanding leaves after 3, 5 and 8 days of magnesium deficiency. The differentially expressed genes (DEGs) were identified by the Exact Test (*p* < 0.01) and categorised into 15 groups based on the day(s) of treatment on which they were differentially expressed.

In search of responsive genes to Mg deficiency, we performed a global transcriptome analysis in mature and expanding leaves on days 3, 5 and 8, and in roots on day 3 after withdrawing Mg from the nutrient solution. Although the shoot growth was inhibited earlier than the root growth (Figures 2A–C), the number of differentially expressed genes (DEGs; *p* < 0.01) on day 3 was larger in roots than in both types of leaves (Table 1). The number of DEGs was larger in expanding than in mature leaves (Table 1), as mirrored by more severe physiological impacts. In expanding leaves, the set of DEGs was not necessarily consistent on different days of treatment; for instance, among the set of up-regulated genes on day 3, only half were also up-regulated on day 5 (Figure 6). This suggests that the gene expression pattern in response to Mg deficiency is unique to the stage of deficiency.

**TABLE 1:** The number of genes differentially expressed (Exact Test, *p* < 0.01) in mature leaves, expanding leaves and roots during magnesium deficiency.

Days of treatment	Mature leaves		Expanding leaves		Roots	
			
	Up-regulation	Down-regulation	Up-regulation	Down-regulation	Up-regulation	Down-regulation
3	54	32	312	146	340	343
5	149	196	2,492	1,951	ND	ND
8	613	23	4,270	2,355	ND	ND

*ND, not determined.*

The DEGs are listed in Supplementary Data S1, and the gene ontology (GO) terms enriched in each DEG set in Supplementary Data S2. On day 3, the GO terms enriched in the set of down-regulated genes in expanding leaves included the light harvesting of photosynthesis (Supplementary Data S2). The down-regulated genes annotated to this term were *CAB2*, *LIGHT-HARVESTING CHLOROPHYLL-PROTEIN COMPLEX I SUBUNIT A4* (*LHCA4*) and *B2.1*, *B6* (*LHCB2.1, B6*) (Supplementary Table S1). The repression of *CAB2* expression was previously reported in Mg-deficient Arabidopsis and proposed to be partly responsible for the chlorophyll degradation (Hermans and Verbruggen, 2005). Besides the repression of *CAB2* expression, the down-regulation of Mg chelatase-encoding genes (Neuhaus et al., 2013) and the up-regulation of genes involved in chlorophyll catabolism (Hermans et al., 2010b; Verbruggen and Hermans, 2013) have also been proposed to be responsible for the chlorophyll degradation. Consistently, both the down-regulation of *MAGNESIUM CHELATASE I1*, *I2* (*CHLI1*, *I2*) and the up-regulation of *NON-YELLOWING 1* (*NYE1*) and *MULTIDRUG RESISTANCE ASSOCIATED PROTEIN 3* (*MRP3*) were detected in expanding leaves on day 5 (Supplementary Table S1), which was before the chlorophyll content decreased (Figure 2D). Besides, we found the down-regulation of a gene involved in chlorophyll synthase (*CHLG*) in expanding leaves on day 5 (Supplementary Table S1). For enzymes involved in carbon fixation, two genes encoding ribulose-1,5-bisphosphate carboxylase/oxygenase small subunit (*RBCS1B* and *RBCS2B*) and a gene encoding phosphoenolpyruvate carboxylase (*PPC2*) were down-regulated in expanding leaves on day 5 (Supplementary Table S1), which may be partly responsible for the reduction in the CO_2_ assimilation rate. Among sucrose/proton symporters (SUCs), *SUC1* was up-regulated and *SUC2* was down-regulated in expanding leaves on day 3 (Supplementary Table S1), when the sucrose content was the same as control (Figure 3C).

For the up-regulated genes in expanding leaves on day 3, the enriched GO terms included: the oxidative stress response, glutathione metabolic process and the regulation of systemic acquired resistance (SAR) (Supplementary Data S2). Genes encoding glutaredoxin (*GRXS13* and *GRX480*) and glutathione S-transferase tau (*GSTU1*, *4*, *8*, *9*, *10*, *19*, *22*, *24* and *25*) were up-regulated within 5 days of Mg deficiency treatment (Supplementary Table S2), suggesting an increase of antioxidative capacity (Hermans et al., 2011). On day 5, genes encoding ferritin (*FER1* and *FER4*) were up-regulated (Supplementary Table S2), consistently with the observations in Mg-deficient rice (Kobayashi et al., 2018). The up-regulated genes annotated to the regulation of systemic acquired resistance are listed on Supplementary Table S2 for discussion.

### Magnesium Deficiency Induced Root Magnesium Uptake Without Transcriptional Regulation of Magnesium Transporter Genes

**FIGURE 7:**
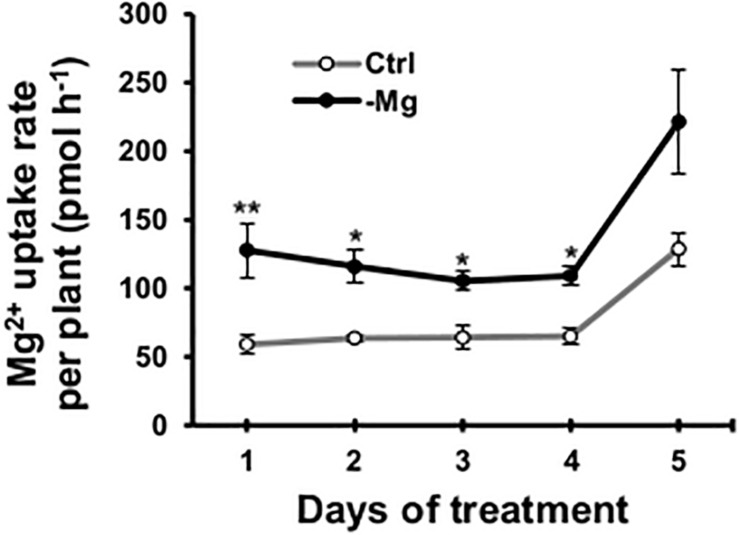
Effect of magnesium deficiency on high-affinity magnesium uptake in roots (^28^Mg tracer study). The root Mg^2+^ uptake rate was determined as the amount of Mg taken up by one plant in 1 h. Data represent means (*n* = 4) with standard error. Ctrl, control, -Mg, magnesium deficiency. Asterisks indicate significant differences in the Mg^2+^ uptake rate between control and Mg-deficient plants (Student’s *t*-test, *p < 0.05, **p < 0.01).

To confirm the early root response to Mg deficiency concerning Mg^2+^ acquisition, we determined the root Mg^2+^ uptake rate in the high-affinity range (50 μM Mg^2+^ in the external solution) using ^28^Mg as a radiotracer. Between days 1 and 4, the high-affinity Mg^2+^ uptake rate was 64–116% (*p* < 0.05) higher in Mg-deficient than in control plant roots (Figure 7). However, none of the known and putative Mg^2+^ transporter genes were differentially expressed in Mg-deficient plant roots on day 3, including genes encoding MRS2 (Schock et al., 2000; Li et al., 2001), prokaryotic Mg^2+^ transporter CorA-like proteins (Smith and Maguire, 1995), MAGNESIUM/PROTON EXCHANGER 1 (MHX1; Shaul et al., 1999), mammalian Mg^2+^ transporter NIPA (Quamme, 2010), NIPA homolog ENOR3 and ENOR3-like proteins (Gao et al., 2018; Supplementary Table S3), suggesting that root Mg^2+^ uptake is controlled by non-transcriptional regulation. Despite the absence of transcriptional regulation of genes encoding putative Mg^2+^ transporters during Mg deficiency, *MRS2* knockout mutants (*mrs2-1*, *mrs2-1* × *mrs2-5*, *mrs2-1* × *mrs2-10*, *mrs2-1* × *mrs2-5* × *mrs2-10* and *mrs2-4*) grown *in vitro* in a Mg depleted solution had altered root morphology compared to the wild type (Supplementary Figure S1).

## Discussion

### Design of Magnesium Deficiency Treatment

To avoid depleting sulphate from the original MGRL solution while imposing Mg deficiency, we substituted MgSO_4_ with Na_2_SO_4_. As the alternative counter cation of SO_4_^2–^, Na^+^ was selected over K^+^ to permit the examination of the effect of Mg deficiency on the K profile. The Na^+^ concentrations in the original and Mg-free MGRL solutions were 2.0 and 5.0 mM, respectively, and the Na content of Mg-deficient plants increased compared to the control plants (data not shown). However, transcript levels of *SALT OVERLY SENSITIVE 1* (*SOS1*), a well-known salt stress marker (Shi et al., 2000) remained unchanged throughout the experiment in leaves and roots (Supplementary Data S1). Therefore, the substitution of Na_2_SO_4_ for MgSO_4_ was considered valid for a Mg deficiency treatment.

### Timeline of Physiological and Transcriptional Responses to Magnesium Deficiency

Sugar accumulation in leaves is regarded as the main cause of chlorophyll degradation observed during Mg deficiency (Tanoi and Kobayashi, 2015). Indeed, many authors documented leaf sugar accumulation before any decline in chlorophyll content or photosynthetic rate in various plant species such as Arabidopsis (Hermans and Verbruggen, 2005), common bean (Fischer and Bremer, 1993; Cakmak et al., 1994a, b), rice (Kobayashi et al., 2013b) and sugar beet (Hermans et al., 2004, 2005). The current study showed that sucrose and glucose accumulate in expanding leaves before any decline in chlorophyll content or CO_2_ assimilation rate (Figures 2D, 3). Oswald et al. (2001) found that transcript levels of *CAB2* were low in the presence of sucrose in an Arabidopsis cell suspension culture. Furthermore, Hermans and Verbruggen (2005) reported that sugar accumulation in leaves could lead to chlorophyll degradation through the repression of *CAB2* expression. However, in the current study, we observed lower *CAB2* transcript levels in expanding leaves on day 3, prior to sugar accumulation (Supplementary Table S1). Besides *CAB2*, transcript levels of *LHCA4* and *LHCB2.1*, *6* also decreased in expanding leaves on day 3 (Supplementary Table S1). This suggests another regulatory component of the expression of these genes than sugar accumulation. This could be an adaptation strategy that reduces electron transport between photosystems to prevent sugar accumulation (Hermans et al., 2004).

Cakmak and Kirkby (2008) proposed that ROS generation during Mg deficiency is likely due to a decrease in CO_2_ assimilation, which causes an over-reduction of the electron transport chain between photosystems. To alleviate the toxicity of ROS, the physiological response to Mg deficiency triggers the activity of antioxidative enzymes and increases the concentration of antioxidant molecules in leaves (Cakmak and Marschner, 1992; Tewari et al., 2006; Hermans et al., 2010b; Kobayashi et al., 2018). The capacity to cope with oxidative stress was recently reported relevant to the tolerance to Mg deficiency in grapevine rootstocks (Livigni et al., 2019). In the current study, the global transcriptome analysis revealed the up-regulation of genes involved in the glutathione metabolic process and the oxidative stress response in expanding leaves (Supplementary Data S2). These transcriptional changes were already evident on days 3, when CO_2_ assimilation had not yet decreased (Figure 3A). Furthermore, genes involved in the reduction process of oxidative stress, such as those encoding thioredoxin superfamily proteins (Arnér and Holmgren, 2000; Marty et al., 2009) and CYP71A23 (Rai et al., 2015) were noticeably up-regulated in roots (Supplementary Data S1). These findings suggest that a decline in net photosynthesis is not the only cause of ROS generation. Previously, Kobayashi et al. (2018) proposed that oxidative stress in Mg-deficient rice is caused by iron toxicity. Here, the up-regulation of genes encoding ferritin (*FER1* and *FER4*), which express in response to iron overload (Gaymard et al., 1996; Petit et al., 2001), was confirmed (Supplementary Table S2). This supports the possible generation of ROS through iron toxicity.

### Induction of Systemic Acquired Resistance in Expanding Leaves During Magnesium Deficiency

The global transcriptome analysis revealed the up-regulation of genes associated with systemic acquired resistance (SAR) in expanding leaves (Supplementary Table S2). The SAR is an adaptive response induced at the whole plant level following a localised exposure to a pathogen (Fu and Dong, 2013). It can be induced through the accumulation of the defence hormone salicylic acid (SA) and secretion of PATHOGENESIS-RELATED (PR) proteins (Fu and Dong, 2013). In expanding leaves, three genes encoding PR proteins (*PR1*, *2* and *5*) were up-regulated within 5 days of Mg deficiency treatment (Supplementary Table S2). Among the other genes up-regulated within the same time window, *ALD1* and *PAD4* act additively to control *PR1* expression and SA accumulation (Song et al., 2004). The PAD4 protein and its interacting partner ENHANCED DISEASE SUSCEPTIBILITY 1 (EDS1) are important activators of SA signalling (Falk et al., 1999; Wiermer et al., 2005), and six other proteins (AED15, EP1, LLP1, PNP-A, PR2 and 5) display EDS1-dependent accumulation (Breitenbach et al., 2014). The up-regulation of these genes suggests that Mg deficiency induces SAR through the activation of SA signalling.

### Nutrient Retranslocation During Short-Term Magnesium Deficiency

During mineral deficiency, plants maintain growth by retranslocating limiting minerals from mature leaves. Maillard et al. (2015) studied the mobility of all essential minerals during leaf senescence in eight crop and tree species and indicated that the mobility of most minerals (P,K,S,Mg,B,Fe,Ni,Cu,Zn,Mo) is variable among plant species. In Arabidopsis, Mg is considered as having limited mobility (Himelblau and Amasino, 2001; Waters and Grusak, 2008). Consistently, symptoms of Mg deficiency tend to appear first in young leaves that develop after deprivation (Hermans and Verbruggen, 2005; Hermans et al., 2010b). In the current study, we found that the Mg content in roots of Mg-deficient plants increased while no additional Mg was available (Figure 4C). This indicates that Mg was retranslocated from mature leaves, where the Mg content decreased during deficiency (Figure 4A). The molecular mechanisms of Mg retranslocation remain unknown. Although some genes of the Mg^2+^ transporter *MRS2*/*MGT* family are expressed in vascular tissues (Gebert et al., 2009), there was no increase in transcript levels of those genes in mature leaves (Supplementary Data S1).

Interestingly, the contents of P,K,B,Mn,Cu,Zn and Mo in mature leaves relatively decreased compared to the control plants within 1 or 2 days following the removal of Mg from the nutrient solution (Figures 4D–F). The contents of these minerals, with the exception of B and Cu, were not affected in expanding leaves or root during 4 days of Mg deficiency (Figures 4D–F). Although the Cu content in expanding leaves could not be determined, it would have been unaffected or increased, based on the increase in Cu content previously observed in Mg-deficient Arabidopsis leaves (Hermans et al., 2010b, 2011). The decrease in contents of P,K,Mn,Cu,Zn and Mo in mature leaves suggests that Mg deficiency either affects the root uptake and xylem transport of these minerals to mature leaves or triggers the retranslocation from mature leaves. The ^32^P radiotracer study revealed that Mg deficiency did not disturb P transport from roots to mature leaves within 6 days (Figure 5), while it reduced the P content in mature leaves within 1 day (Figure 4D). Furthermore, 4 days of Mg deficiency noticeably reduced P transport from roots to expanding leaves (Figure 5), while it did not affect the P content in expanding leaves (Figure 4E). These findings indicate that the decrease in mineral contents in mature leaves is not attributed to the affected root uptake and xylem transport but to the stimulated retranslocation. Previously, it was shown that P,K,Cu,Zn (Himelblau and Amasino, 2001; Waters and Grusak, 2008) and Mo (Himelblau and Amasino, 2001) were retranslocated from senescing leaves of Arabidopsis. Therefore, the retranslocation of these minerals during Mg deficiency implies the accelerated senescence of mature leaves. Calcium (Biddulph et al., 1958) and Mn (Hocking et al., 1977) are recognised to have low phloem mobility. There was no evidence of Ca retranslocation from Arabidopsis leaves in either the current or previous studies (Himelblau and Amasino, 2001; Waters and Grusak, 2008). The only contradiction between the current and previous observations is that Mn was retranslocated from Mg-deficient mature leaves (Figure 4D) while it was not from senescing leaves of Arabidopsis (Himelblau and Amasino, 2001; Waters and Grusak, 2008). The retranslocation of Mn has been observed in some species such as barley, bread wheat (Maillard et al., 2015), sour orange and sweet orange (Papadakis et al., 2007). For Mn retranslocation, Arabidopsis may have a common mechanism to those species, which is triggered by Mg deficiency but not during leaf senescence.

## Data Availability Statement

The raw reads and normalised count data in the global transcriptome analysis can be found in NCBI’s Gene Expression Omnibus (https://www.ncbi.nlm.nih.gov/geo/query/acc.cgi?acc= GSE140070).

## Author Contributions

TO, NK, and KT designed the experiments. TO conducted most of the experiments, analysed the data and wrote the manuscript. CH conducted the root morphology analysis and helped analyse the data. YI, AS, and KS assisted in the global transcriptome analysis. NA assisted in sugar quantification. RS and TOga assisted in the ^14^C radiotracer study. HS, RI, TN, and KT produced and purified ^28^Mg. NK, CH, and KT supervised the writing of the manuscript. All the authors approved the final version of the manuscript.

## Conflict of Interest

The authors declare that the research was conducted in the absence of any commercial or financial relationships that could be construed as a potential conflict of interest.
