# Supplementary material for: Short-Term Magnesium Deficiency Triggers Nutrient Retranslocation in Arabidopsis thaliana
Source: Front Plant Sci. 2020 Jun 4;11:563. doi: 10.3389/fpls.2020.00563 (PMC7287120; doi:10.3389/fpls.2020.00563)
Supplement: Supplementary file 4 [file Image_1.pdf]

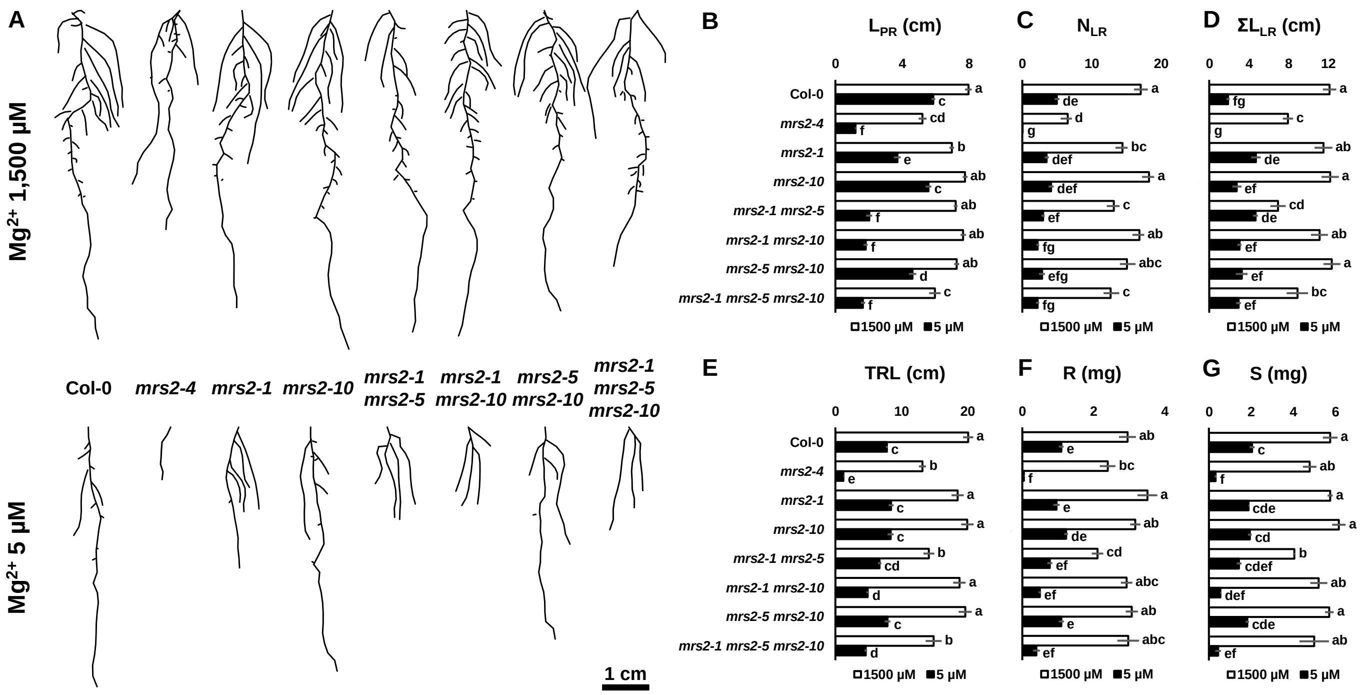

SUPPLEMENTARY FIGURE S1 | Root system architecture and biomass production of wild type and *MRS2* mutant lines. Seedlings were grown *in vitro* on vertical agar plates with sufficient (1,500 µM) or low (5 µM) Mg supplies, and root organs were scanned 10 days after germination. **(A)** Representative root systems of different genotypes grown with sufficient (top) or low (bottom) magnesium supplies. Scale bar: 1 cm. **(B-E)** Root morphological parameters: length of primary root (L_{PR}) **(B)**, number of lateral roots longer than 1 mm (N_{LR}) **(C)**, sum of lateral root lengths (ΣL_{LR}) **(D)** and total root length **(E)**. Data represent means (n = 13 – 38) with standard error. **(F,G)** Biomass production parameters: root **(F)** and shoot **(G)** fresh weight per plant. Data represent means (n = 3 – 6) with standard error. Different letters indicate significant differences in measured variables (a factorial ANOVA, $p < 0.05$).
